# New Onset of Linear Purpura on the Back: Coining Therapy-Associated Ecchymoses

**DOI:** 10.7759/cureus.8833

**Published:** 2020-06-25

**Authors:** Adrija K Darsha, Philip R Cohen

**Affiliations:** 1 Medicine, University of California San Diego, La Jolla, USA; 2 Dermatology, San Diego Family Dermatology, National City, USA

**Keywords:** coin, coining, ecchymoses, erythema, pain, spoon, spooning

## Abstract

Coining therapy is a treatment commonly used in complementary and alternative medicine. The practice has its origins in several different Asian countries. It is used to treat numerous conditions, such as chronic pain, fever, flu, headaches, heatstroke, and upper respiratory infections. Coining is performed by vigorously rubbing a rounded instrument following the application of lubricant to the affected area. Hence, patients who have undergone coining therapy frequently present with macular erythema, petechiae, and/or raised ecchymoses at the sites of treatment. The cutaneous sequelae following treatment with coining on a Vietnamese man are described. Ecchymoses caused by coining usually resolve spontaneously within one to two weeks. While coining is generally regarded as a safe practice, mild or - albeit rarely - more severe complications may occur. Therefore, this procedure is contraindicated in certain patients including those with bleeding disorders, Von Willebrand disease, or those taking antiplatelet or anticoagulant medications. Several randomized-control studies suggest coining to be an effective treatment for chronic neck and lower back pain. Immediate pain relief at the treated site may result from increased circulation; thus, the venting of heat may mitigate the effects of the inflammation and pain. However, much remains to be learned about the mechanisms of longer-term pain relief in coining therapy. The use of complementary and alternative medicine techniques such as coining has increased in the United States; therefore, clinicians’ evaluation and management of their patients would benefit from an understanding of the individual’s sociocultural practices and health beliefs.

## Introduction

Coining is a treatment used in complementary medicine. It originated in Southeast Asia. Numerous medical conditions can be treated with this technique [1].

In East Asian medicine, pain is seen as a form of stasis. Common myalgias are thought to result from the stress of repeated activity, sustained posture, or changes in temperature. If the pain resolves from touch or movement, it is referred to as Qi stasis; if the pain persists or returns to a particular location, it is called Qi and blood stasis. Coining is thought to release “heatiness” or “negative energies” from the body, and is thus used as a therapeutic intervention for pain and pathology manifesting Qi and blood stasis [2,3].

The cutaneous sequelae following the treatment with coining of a Vietnamese man are described. The salient features of this therapeutic alternative are discussed. Additional inquiry of patients in whom coining therapy is suspected should be performed to exclude other etiologies such as bleeding disorders, child or elder abuse, hemostasis altering medications, and trauma [2,3].

## Case presentation

A 45-year-old Vietnamese man returned for a follow-up visit regarding his folliculitis and dermatitis. The day prior to his appointment, he experienced fever, soreness of the throat, and neck stiffness of less than 24 hours of duration. He sought treatment from his family doctor.

**Figure 1:**
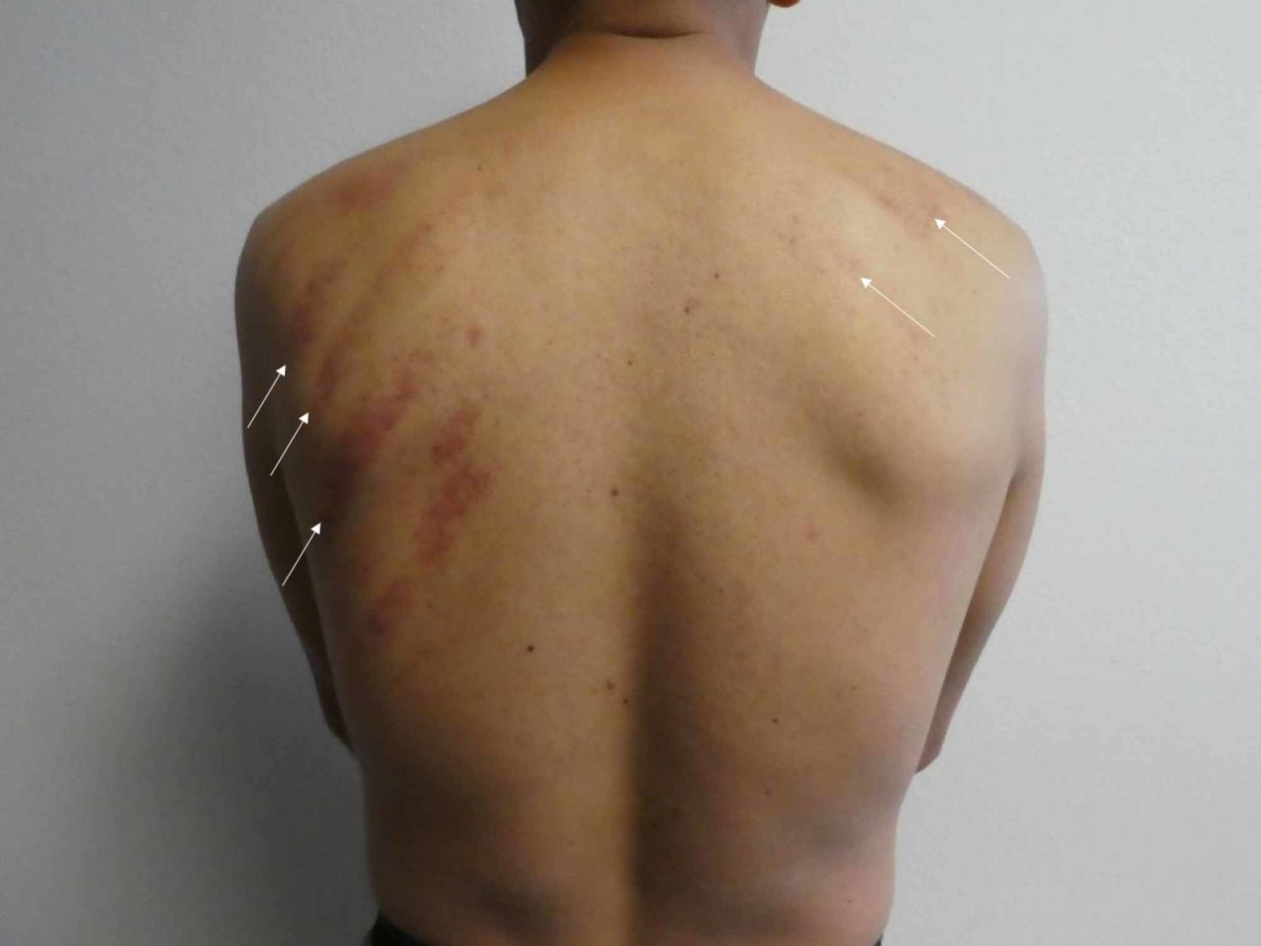
Coining therapy-associated ecchymoses. A distant view of the back of a 45-year-old Vietnamese man shows coining therapy-associated linear ecchymoses (white arrows) on the skin overlying the left and right scapula.

**Figure 2:**
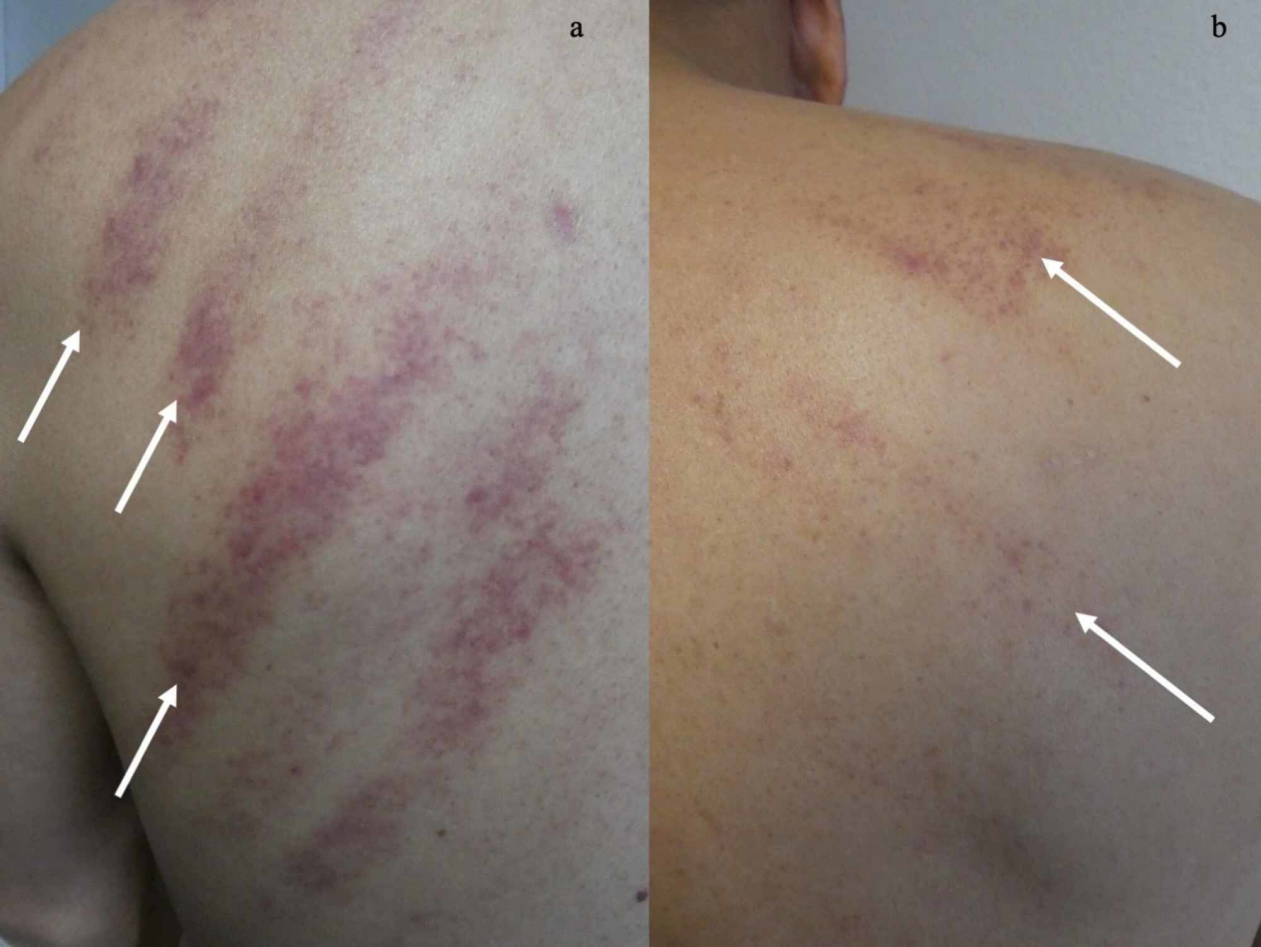
Linear purpura resulting from coining on the upper back. Closer views of the left (a) and the right (b) side of the upper back show linear ecchymoses (white arrows) that occurred as a sequela of coining therapy. The pattern of linear purpura resembles that of a pine tree on the patient’s left upper back.

Cutaneous examination of his back showed linear purpura in a pine-tree-like pattern on his left and right scapula (Figures 1, 2). He was not taking any antiplatelet or anticoagulant medications. He did not have a bleeding disorder. 

The pronounced ecchymoses prompted additional inquiry regarding their development. The family doctor had vigorously treated his presumed viral upper respiratory tract infection by vigorously rubbing the areas of his back overlying the lungs with a round smooth coin.

Correlation of the clinical history and lesion morphology established the diagnosis of coining therapy-associated ecchymoses.

Following the coining procedure, the patient took 650 milligrams of acetaminophen and went to sleep. By the following morning, his symptoms had resolved. At the subsequent office visit one month later, all of the coining-associated ecchymoses had resolved.

## Discussion

Coining has its origins in several different Asian countries. Each location has its own designated nomenclature (Table 1) [1,2,4,5]. The term *gua sha* in Chinese translates to “scraping sand” [1]. Similarly, the term *cao gio* in Vietnamese translates to “scratch out the wind” [2].

**Table 1:** Nomenclature for coining

Location	Name	References
Cambodia (Khmer)	kos kyal	[2]
China	gua sha	[1,2,4 5]
Indonesia	kerik	[2]
Laos	khoud lam	[2]
Vietnam	cao gio	[1,2,4,5]

Coining has been used to treat numerous conditions (Table 2) [1,2,4-9]. These not only include upper respiratory infections and headaches, but also flu, fever, heatstroke, and chronic pain [2]. Our patient experienced prompt and sustained resolution of all his symptoms within 12 hours of the procedure.

**Table 2:** Indications for coining

Indication	References
Acute pain	[2]
Chills	[4]
Chronic pain	[2,6-8]
Colds	[2,9]
Cough	[5]
Digestive disorders	[1,6]
Fevers	[1,2,4-6,9]
Flu	[2,9]
Headache	[1,4,5]
Heatstroke	[2,6,9]
Musculoskeletal problems (fibromyalgia, severe strain, spasm, or injury)	[1,2,6,9]
Respiratory problems (asthma, bronchitis, and emphysema)	[2,6,9]
Seizures	[4]
Sore throat	[5]
Upper respiratory infections	[1,5]
Vomiting	[4]

Coining is performed using either a coin or similarly shaped object, such as a spoon [2]. Prior to vigorously rubbing the instrument on the affected area, lubricant is applied. Our patient had Tiger Balm, an ointment used for pain relief, applied prior to the coining procedure.

The coining treatment then begins by pressing a smooth, rounded edge into the flesh enough to contact the fascial layer, but not so hard that it causes pain or discomfort. Stroking, beginning at the center line, is repeated in one direction until significant petechiae, preferably without extensive ecchymoses, appears. Coining is continued at adjacent stroke lines until the area to be treated is covered, taking approximately five to seven minutes [2].

The forceful rubbing experienced during coining by the patient is considered to be necessary to promote the resolution of symptoms. Therefore, the procedure tends to be painful. However, these symptoms usually resolve shortly after the procedure has been completed.

The vigorous rubbing usually results in macular erythema, petechiae, and/or raised ecchymoses at the sites of treatment. Therefore, this procedure is contraindicated in patients with bleeding disorders, Von Willebrand disease, or those taking antiplatelet or anticoagulant medications [3].

The resulting ecchymoses caused by coining usually resolve spontaneously within one to two weeks. However, albeit uncommon, mild or more severe complications may occur (Table 3) [2-5,10,11].

**Table 3:** Adverse events associated with coining

Adverse event	References
Allergic nickel dermatitis	[10]
Burns (partial and full thickness)	[2,4,5,11]
Camphor toxicity	[2-5]
Cerebellar hemorrhage	[2,4,5]
Contact dermatitis	[4]
Hematuria (microscopic or mild)	[2,4]

The clinical differential diagnosis includes bleeding disorders and trauma. Indeed, the morphology of the lesions can mimic either child abuse or elder abuse. Clinicians unaware of the etiologies for the presentation of lesions may contact child or adult protective services prior to establishing the correct etiology for the lesions [2,3].

Since coining causes bruises, lesions should not be blanchable. Splenomegaly and hepatomegaly may be the source of underlying issues with ecchymoses, and thus should be considered in the differential. Additionally, dengue hemorrhagic fever should be considered in regions where it is endemic, as the resulting ecchymoses can be similar to coining [3].

Consistent with the traditional East Asian perspective that coining moves blood, microcirculation is locally increased fourfold for the first seven and one-half minutes and remains significantly elevated for at least 25 minutes following treatment. Therefore, immediate pain relief at the treated site may result from the increased circulation; thus, the venting of heat may mitigate the effects of the inflammation and pain. Despite this partial knowledge of coining physiology, the definitive mechanism for the longer-term pain relief, especially at sites that do not experience increases in microcirculation, remains to be elucidated [2,9].

## Conclusions

Coining is an alternative medicine treatment originating in Southeast Asia. It is utilized as a primary or adjuvant therapy for several conditions, such as chronic pain, fever, flu, and upper respiratory infections. Although coining therapy is generally a safe treatment with little risk for serious side effects, it can be associated with adverse events such as camphor intoxication, contact dermatitis, hematuria, minor burns, or rarely, cerebellar hematoma with herniation. Coining-associated skin lesions can present as petechiae and ecchymoses which can mimic child or elder abuse. The lesions commonly resolve spontaneously; however, post-inflammatory hyperpigmentation may occur.
